# Teleradiology and technology innovations in radiology: status in India and its role in increasing access to primary health care

**DOI:** 10.1016/j.lansea.2023.100195

**Published:** 2023-04-14

**Authors:** Anuradha Chandramohan, Viswajit Krothapalli, Ann Augustin, Madhavi Kandagaddala, Hannah Mary Thomas, Thambu David Sudarsanam, Ammar Jagirdar, Shalini Govil, Arjun Kalyanpur

**Affiliations:** a Department of Radiology, Christian Medical College, Vellore, 632004, India; b Christian Medical College, Vellore, 632004, India; c Department of Clinical Epidemiology, Christian Medical College, Vellore, 632004, India; d Products, Qure-AI, Mumbai, India; e Naruvi Hospital, Vellore, India; f Pun Hlaing Hospital, Myanmar; g Teleradiology Solutions, Whitefield, Bengaluru, Karnataka, 560048, India

**Keywords:** Teleradiology, Mobile-teleradiology, Primary health care, Access, Technology, Artificial intelligence, Universal health coverage

## Abstract

**Background:**

There is an inequitable distribution of radiology facilities in India. This scoping review aimed at mapping the available technology instruments to improve access to imaging at primary health care; to identify the facilitators and barriers, and the knowledge gaps for widespread adaptation of technology solutions.

**Methods:**

A search was conducted using broad inclusive terms non-specific to subtypes of medical imaging devices or informatics. Work published in the English language between 2005 and 2022, conducted primarily in India, and with full manuscripts were included. Two authors independently screened the abstracts against the inclusion criteria for full-text review and a senior author settled discrepancies. Data were extracted using DistillerSR software.

**Findings:**

43 original articles and 52 non-academic materials were finally reviewed. The data was from 10 Indian states with n = 9 from rural settings. The broad trends in original articles were: connectivity using teleradiology (n = 7), mobile digital imaging units (n = 9), artificial intelligence (n = 16); mobile devices and smartphone applications (n = 7); data security (n = 7) and web-based technology (n = 2); public-private partnership (n = 9); cost (n = 2); concordance (n = 19); evaluation (n = 4); implementation (n = 2).

**Interpretation:**

Available evidence suggests that teleradiology when combined with AI and mobile digital imaging units can address radiologist shortages; strengthen programs aimed at population screening and emergency care. However, there is insufficient data on the scale of teleradiology networks within India; needs assessment; cost; facilitators, and barriers for implementation of technologies solutions in primary healthcare settings. Regulations governing quality standards, data protection, and confidentiality are unclear.

**Funding:**

The authors are The Lancet Citizen's Commission fellows. The Lancet Commission has received financial support from the Lakshmi Mittal and Family South Asia Institute, 10.13039/100007229Harvard University; 10.13039/501100005918Christian Medical College, Vellore (CMC), Vellore; Azim Premji Foundation, Infosys; Kirloskar Systems Ltd.; Mahindra & Mahindra Ltd.; Rohini Nilekani Philanthropies; and Serum Institute of India. The views expressed are those of the author(s) and not necessarily those of the Lancet Citizens' Commission or its partners.

**Research in context**

**Evidence before this study**

We searched PubMed and Google Scholar with no date restrictions in the first week of March 2021, for scoping and systematic reviews from India on teleradiology, mobile teleradiology, mobile imaging units, and artificial intelligence in improving access to primary health care published in the English language. We used the terms ([“teleradiology” OR “mobile teleradiology” OR “artificial intelligence” OR “AI” OR “mobile X-ray” OR “mobile digital X-ray”] AND [“primary health care” OR “primary health” OR “PHC”] AND [“access” OR “treatment]). To our knowledge, no study or review article modeled the impact of teleradiology and the new technologies in improving access to imaging care in primary healthcare in India.

**Added value of this study**

This scoping review provides an overview of the available instruments that can potentially improve access to imaging care for the rural population of India and their current state; addresses some of the knowledge gaps in the published literature, and highlights others where more work needs to be done.

**Implications of all the available evidence**

The available evidence suggests that technologies such as teleradiology, artificial intelligence-based algorithms, and mobile digital imaging units can address healthcare provider shortages and improve access to health. The current work highlights the knowledge gaps in the quality standards, cost-effectiveness, facilitator and barriers for implementation, and the evaluation of teleradiology and other technologies in a primary healthcare setting. Given the evolving regulations on data protection and the currently immature digital health system in India, the findings of our scoping review will be useful in shaping these efforts.

## Introduction

Radiological investigations help with an accurate diagnosis, risk stratification, and prognostication of diseases. This positively influences treatment decisions, outcomes, referral pathways, and health resource allocation. However, setting up a radiology service is resource intensive. Sixty percent of our population live in rural areas and their access to health care, high-quality diagnostics, and specialist opinion remain difficult. There is an inequitable distribution of radiology facilities in the country with most of the trained radiologists being concentrated mainly in larger cities and towns. There are too few radiologists for a population of 1.4 billion. For a long time now, teleradiology has been one of the effective solutions for bridging these gaps between demand and supply. There is an increased appetite for technology adoption by radiology services across the country to meet the demands placed on them. Picture archiving and communication systems (PACS) and radiology information systems (RIS) have tremendously increased the efficiency, productivity, and throughput of imaging services. Improved connectivity through internet and mobile technology, teleradiology, artificial intelligence, developments in medical imaging informatics, and portable or mobile medical imaging devices have the potential to enable remote locations within the country to gain access to better health care and diagnostics. Though we saw examples and use case scenarios in action demonstrating the value of these during the COVID-19 pandemic, there is little published literature on the degree to which technologies including teleradiology have reached the last mile, and their influence in improving access to health at primary healthcare level is sparse and incomplete.

### Aims and objectives

This review aims to provide an overview of teleradiology and technology solutions available in India. In this review, we will report on the existing instruments in India technology tools, policy tools, and Public-Private Partnerships (PPP), and how they have enabled access to imaging care and diagnostics at the primary healthcare level.

#### Primary objective

i) To identify and consolidate the literature available on the technology instruments that have enabled access to imaging care at the primary health care level.

#### Secondary objectives

i) To map the barriers and facilitators for adopting technology solutions to improve access to imaging care at the primary health care level.
ii) To identify knowledge gaps and missing links to use technology solutions to improve access to imaging care at the primary health care level.

## Methods

The protocol for the scoping review was structured using the Preferred Reporting Items for Systematic Reviews and Meta-Analyses extension for scoping reviews (PRISMA-ScR) checklist.^1^

### Protocol design

The Arksey and O'Malley methodological framework was used to perform this scoping review primarily to identify key concepts, rapid knowledge synthesis, and to identify the research questions.^1^ The target group included healthcare providers, patients/consumers, policymakers, and co-researchers to better understand the topic and its relevance for re-imagining healthcare and its delivery. The following steps were performed for completing the scoping review: (a) Identify the research question (b) Finalize the operational definitions (c) Identify relevant studies (d) Select the studies based on the inclusion and exclusion criteria (e) Chart the data and (f) Collate, summarize, and report the results.^2^

#### Identify the research question

Interpretation of radiological images for diagnosis and further recommendation requires highly trained personnel. However, such skilled health personnel are concentrated in large cities and urban areas and are very few in number in semi-urban and rural areas even in high-income countries.^3^^,^^4^ Studies have shown that teleradiology plays an important role in bridging this gap and improving access to healthcare in remote geographical settings. However, very little is known about the influence of teleradiology practices and new technologies in India. This knowledge gap was the motivation for this scoping review. The primary and secondary objectives of this scoping review are mentioned in Table 1.

**Table 1:** Scoping review of primary and secondary research questions.

Primary research question	Secondary research questions
1 What are the teleradiology, mobile teleradiology, and other digital technologies used to improve access to imaging care at the primary health care level?	1 What are the risks/disadvantages associated with teleradiology and technology solutions in primary health care? 2 What are the benefits/advantages associated with teleradiology and technology solutions in primary health care? 3 What are the knowledge gaps in the widespread adaptation of technology solutions to deliver imaging care in primary health care settings?

### Stages of the review

#### Identify the relevant studies

The search for relevant studies was conducted using the following electronic databases: PubMed, Google Scholar, IndMed, Cochrane databases of systematic reviews, Scopus, and Web of Science. Unpublished reports from these sources including but not limited to reports from the government, teleradiology service providers, etc. were also included. The search strategy (included in Appendix1) was adapted for each database/source and changes were made to the research question to account for the changes included in the review process. Since Radiology is a rapidly evolving specialty, we chose to limit the data search to between 2005 and 2022. We only reviewed English language studies, primarily from India. We however did not exclude material the based on study design. As a supplementary search strategy, we explored the references of the full-text references, which were included in the review for additional material.

#### Study selection

We undertook a three-phase screening of the literature by four qualified reviewers. The inclusion and exclusion criteria to select the relevant literature and the operational definitions for terms such as teleradiology, medical imaging informatics, and deep or machine learning are provided in Table 2. DistillerSR (Evidence Partners, Ottawa, Canada) software was used to manage the records and the data. In the first phase of the review, the title and the abstract of all studies were screened for relevance by two reviewers who ensured that all relevant articles were included as per the search strategy. In the second phase of the review, each full-text article that was cleared in the first phase was further screened for specified inclusion and exclusion criteria by any two of the reviewers. The data was extracted from each screened study by one of the two reviewers involved in full text screening and cross-checked by the second reviewer. Discrepancies were resolved after a discussion with an independent reviewer.

**Table 2:** Inclusion & exclusion criteria and operational definitions.

**Inclusion criteria**	
Population	Population/individuals of a community/region/state seeking healthcare and people/organization/institutions/agencies delivering health care.
Intervention	Teleradiology, medical imaging informatics, and technology
Comparator	All studies using teleradiology facilities and medical imaging informatics and technology
Outcome	Equitable health care/efficient utilization of available resources/reduced waiting time for diagnosis/access to a specialist's opinion.
Study Type	1 Any type of study; experimental (randomized controlled trials (RCTs), quasi-RCTs, non-RCTs), observational (cohort, case–control, cross-sectional), and review (systematic review, meta-analysis scoping review) studies. 2 Relevant published literature, including reports, policy documents from the government, audits, etc from the year 2010 onwards that report the latest developments in the field. 3 Published in the English language.
**Exclusion Criteria**	
	Studies that did not use teleradiology/informatics-based technologies used in imaging/for health care accessibility will be excluded.
**Operational definitions**	
Teleradiology	Teleradiology is the electronic transmission of diagnostic radiographic imaging studies between two geographical locations for interpretation and/or consultation.
Deep learning/Machine learning	Machine learning and deep learning are branches of artificial intelligence and include techniques and algorithms that enable computers to discover complicated patterns in large data sets; in this case medical images.
Medical Imaging Informatics	Medical Imaging informatics refers to the application of information technology to medical imaging to provide healthcare services. It includes but is not limited to image creation and acquisition, storage and retrieval, image processing and analytics, visualization of images, reporting, and communications.

#### Charting the data

A customized data extraction form was created on DistillerSR, following pilot testing by the research team. The following data were extracted from the included studies: source, year of publication, title, authors, study type, demographic details, aims/purpose, methodology, sample size, study duration (if applicable), intervention type, comparator (if applicable), technology, type of disease on this which was undertaken, study outcome and salient findings related to the review question. An appropriate quality-assessment tool was used to assess the overall quality of the reported studies. We reviewed and included the grey literature to fill in the specific gaps in the white literature.

Qualitative methods were used to summarize and report the key findings of the studies included in the scoping review under the outcomes listed and defined in Table 3.

**Table 3:** Outcomes and their definitions.

Connectivity	Methods of connecting various stakeholders providing or seeking health interventions such as patients, image sources, radiologists, clinical referrers.
Image capture/display and image transfer	Methods of acquiring, displaying, and transferring radiology images from the image source to the reporting stations of the radiologists.
Data security	Methods of protecting digital healthcare-related data from unauthorized access, theft or data alteration or corruption.
Shared interoperability	Exchanging or sharing healthcare-related data in a secure manner between the stakeholders. E.g., Public-private partnerships, and data sharing between computers using local area networks (LAN) and wide area networks (WAN).
Concordance	Studies that describe the comparison of the diagnostic accuracy or the degree of agreement between newer technologies or and traditional methods.
Needs assessment	Systematic procedures that are undertaken to assess the gaps between the current and the desired state.
Cost	Cost of various processes involved in using teleradiology and other imaging-related technologies to improve access to health care. Cost of clinical interventions with and without the use of teleradiology/mobile radiology and newer technologies.
Evaluation	Systematic gathering of information to assess the performance of various processes involved in using teleradiology and other imaging-related technologies to improve access to health care.
Implementation/	Strategies promoting the uptake of research findings at ground level to enhance the adoption of clinical intervention.

### Role of funding source

None.

## Results

### Identification of studies and characteristics of included studies

**Fig. 1:**
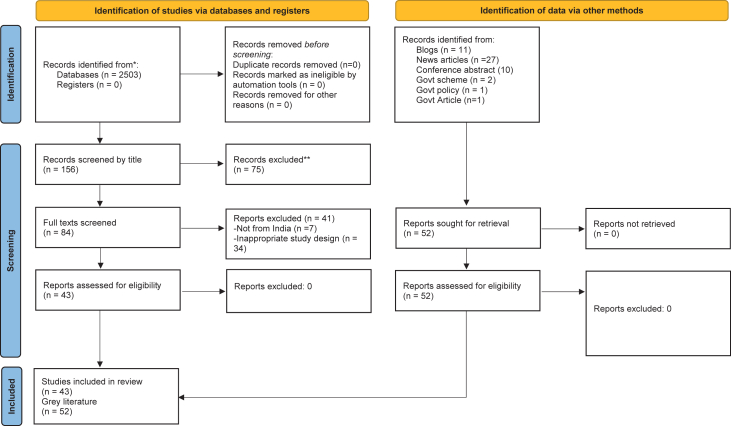
Flowchart of included studies.

Initial screening of the literature based on title yielded 156 articles. Of these 156, a total of 84 potentially eligible publications required the full text version for further investigation. After a review of the full-text version, a total of 41 reports were excluded either because they were not relevant to this review (n = 34) or because these studies that were not from India (n = 7). The remaining 43 publications fulfilled the inclusion criteria. Of the 43 included, there were n = 19 cross-sectional studies, that included prospective (n = 10) and retrospective (n = 9) studies. Other studies included qualitative studies (n = 3), and editorial pieces or commentaries (n = 5). The study design was not mentioned in some studies (n = 16) included. We used the Critical Appraisal Skills Programme (CASP) checklist to evaluate the quality of the studies. The quality of studies selected for review was heterogeneous with the majority being average to low in quality. A total of 52 articles that can be categorized as grey literature and fulfilled the inclusion criteria were considered for analysis. These included blogs (n = 11); news articles (n = 27); conference abstracts (n = 10); policies and articles published by the government (n = 2) and government schemes (n = 2). A summary of the selection process is presented in Fig. 1.

### Demographic data from included studies

Studies included for review emerged from ten different states of India with a state rank in development ranging from 2 to 18, and a state health index ranging from 44.6 to 79. Of these, only nine studies (21%) were from the rural setting and four studies were from an urban setting. In the remaining studies, the study setting was either not mentioned or not applicable due to the nature of the study. The top diseases that were studied include tuberculosis, pneumonia, intracranial bleeding, emergency radiology, lung cancer, and breast cancer. Of these, 20% of the studies were on tuberculosis. The sample size of the studies had a wide range from six to 2.5 lakhs, and similarly, the number of imaging studies included in various studies were between 8 and 2.5 lakhs examinations. The top imaging modalities featured in the included studies were chest radiographs, mammography, and computed tomography of the brain. The majority of the included studies were reported for the adult population. Only four studies included paediatric patients aged between 15 and 17 years, and two studies included the elderly (>65 years).

### Key moments in imaging care at the primary healthcare level

The key moments in the imaging care of rural patients in India are access to machines, digitization of analogy films, image transfer and imaging viewing, teleradiology, artificial intelligence help, data security, the role of physicians, and operational challenges. These key moments are discussed in the following paragraphs.

#### Mobile digital imaging units

Mobile digital X-ray units have been recognized as an important method of bridging access gaps in rural areas of India. Mobile health vans equipped with basic diagnostic facilities (X-ray, ultrasound), basic lab tests, and a mini clinic were launched as a means of providing access to primary healthcare services in difficult-to-reach terrains with poor road connectivity. The feasibility, the positive role of mobile digital X-ray units in increased detection, and decrease in the time to diagnosis, diagnostic accuracy, and the cost of adding mobile digital X-ray units in the point of care diagnosis of pulmonary tuberculosis and its value in diagnosing sputum-negative tuberculosis has been studied in rural Haryana. These studies were conducted in a PPP model integrating the mobile digital X-ray service provided by a private corporate hospital with the national tuberculosis program in that area.^5^, ^6^, ^7^, ^8^ Two preliminary studies showed the cost-effectiveness of using mobile digital X-rays for the detection of pulmonary tuberculosis in rural India.^9^^,^^10^ In some states, the private sector has taken the lead in launching such mobile health vans. One such example is the Uttaranchal Mobile Hospital and Research Center (UMHRC), which is a three-way partnership among the Technology Information, Forecasting and Assessment Council (TIFAC), the Government of Uttaranchal, and the Birla Institute of Scientific Research (BISR). The motive behind the partnership was to provide health care and diagnostic facilities to poor and rural people at their doorstep in the difficult hilly terrains. TIFAC and the State government shared the funds sanctioned to BISR on an equal basis.^11^

Another use case scenario demonstrating the feasibility of mobile imaging units is the breast cancer screening usinga mobile digital mammography un it.^12^ However, this study reports the challenges in maintaining the quality of mammography and the gaps in the training of healthcare workers.^12^ In summary, the above examples demonstrate the feasibility and usefulness of mobile digital X-ray units for providing imaging care to remote geographies of India. But the success of these programs will depend on the availability of trained personnel and quality checks.

#### Conversion of analog to digital X-ray units

Improved accuracy, less radiation dose, improved workflow, and better integration with PACS and teleradiology have resulted in a worldwide transition from conventional screen film radiography to digital X-ray units. Over the past decade, we have seen a similar steady increase in the installations of digital X-ray units in India. However, this has mainly been the large government hospitals, corporate hospitals, and private nursing homes. Most primary healthcare centers (PHC) in the country still have analog X-ray units.^13^ PHCs usually do not have a radiologist. There is often a delay in the diagnosis, which is closely linked to these factors. Thus, there is interest in technology that can convert analog X-rays films to digital images and those that allow upgrading analog X-ray units to digital imaging units. We found one study published in 2013, which reported a scalable and low-cost method of converting analog X-rays to digital images with non-linear image enhancement using an android application.^13^ One study reports the use of megapixel digital cameras for digitizing the analog images acquired on legacy analog systems in PHC settings in Tripura, a north eastern state of India.^14^

#### Image viewing and image display methods

Low-cost alternative image display units have been studied to assess the feasibility and as a method to facilitate quick diagnosis. A concordance study with a sample size of 400 compared the agreement between two experienced readers in the interpretation of chest X-rays displayed on picture archiving and communication systems (PACS) and WhatsApp messenger.^8^ This study demonstrated excellent to a substantial agreement for the interpretation of normal chest X-rays, pneumonia, and active and old tuberculosis. But there was little agreement for diagnosis made on subtle imaging findings such as cancer, pleural effusion, and hilar nodes. Similar attempts have been made in the diagnosis of maxillofacial trauma using emails and instant messaging platforms such as WhatsApp to enable remote oral radiology consultations.^15^ Another concordance study assessed the diagnostic accuracy of detecting intracranial bleeding on CT scans viewed on laptops and netbooks and demonstrated the feasibility of using such alternative mobile display units to enable quick access to imaging diagnosis time-sensitive clinical areas like emergency medicine.^16^ Available evidence shows that mobile image displays on smartphone applications like WhatsApp and netbooks may be used for point-of-care diagnosis in emergency and trauma settings. But workstations with high-resolution monitors will still be required for accurate diagnosis of subtle and complex abnormalities.

Cloud/web-based technology has been of interest for bringing gaps in the connectivity. eSanjeevani program, a national teleconsultation service is being prepared for a national roll-out at 155,000 health and wellness centres across rural India by the Ministry of Health and Family Welfare (Government of India) under the Ayushman Bharat Scheme, which is a health insurance scheme. eSanjeevani aims to connect multiple Health and Wellness Centres (HWC) in rural India with secondary and tertiary level hospitals.^17^ The eSanjeevani program had researched the free web-based DICOM image viewers and recommended six of them for national teleconsultation services.^17^ Collaborative Digital Diagnosis System (CollabDDS) is another web based digital image viewing and sharing platform funded by Ministry of Communications & Information Technology, New Delhi and developed by the National Knowledge Network. The purpose of this platform is to enable communication between healthcare providers in the rural areas and expert radiologists by sharing digital images.^18^ Another successful collaborative effort using a cloud-based platform is by the Koita Center for Digital Oncology (KCDO) established by the National Cancer Grid (NCG) that connects the oncology centres with different levels of expertise and resources.^19^ Through this platform, weekly or fortnightly Multi-Disciplinary Team (MDT) meetings are conducted with the remote and peripheral cancer hospitals to enable clinical decision-making and to help referral pathways to hospitals with specific oncology expertise. The above examples show that the rural population can gain access to diagnostic expertise using cloud-based technologies. This can potentially decrease the out-of-pocket expenditure and the loss of wages incurred by the time, money, and effort spent on accessing diagnostics.

#### Teleradiology

Teleradiology practice in India dates back to 1996 when the first successful use of teleradiology was demonstrated by Jankharia Imaging, a Mumbai-based private imaging center where images were transferred between the scan center and the radiologist's homes mainly to deliver emergency radiology reports.^20^ Subsequently, there were public demonstrations of the capabilities of teleradiology by imaging companies such as Siemens and Wipro GE in 1997. The first teleradiology company, Teleradiology Solutions was set up in 2002 in Bengaluru by Dr. Arjun Kalyanpur and his colleagues where US board-certified radiologists read scans for hospitals in the USA.^20^ The company subsequently extended its services to Singapore and India. Following this, Wipro Technologies provided night-time reporting services and 3D reconstruction services.^20^ Ever since, there has been a boom in teleradiology industry in India. Though the exact data on the number of teleradiology companies and their network is not readily available in published literature at the time of this review, a google search of “Teleradiology companies in India” returned 1,26,000 results, which speaks for itself. The boom of the teleradiology industry in India was born out of the need to address shortages in radiologists; lack of radiology services and personnel in small towns and rural India; the demand for round-the-clock radiology services; the need for shared expertise and second opinions; need for better utilization of manpower and resources; and the perceived potential of teleradiology to address many of these issues. Though there are no formal studies these facts have been highlighted repeatedly by subject matter experts.^14^^,^^20^, ^21^, ^22^, ^23^

The first example of teleradiology service to the rural population in India was reported by Char et al., in 2010.^24^ In this work, authors describe the teleradiology service provided to Ramakrishna Mission Hospital in Itanagar, Arunachal Pradesh, which catered to the tribal population in the area and did not have an on-site radiologist. Teleradiology service was provided by a commercial teleradiology provider located in Bengaluru, Karnataka to a remote hospital 3000 km away for a one-year duration. Following a short training through remote access, two onsite radio technicians were able to upload and transfer images as compressed ZIP and provide clinical and demographic details of the patients using a radiology information system (RIS) with the help of a simple broadband connection via Asymmetric Digital Subscriber Line (ADSL) and a secure File Transfer protocol (sFTP). Authors have reported a turnaround time of 30 min for emergency radiology studies and 6 h for non-emergency or elective studies.^24^ This example showed the feasibility of teleradiology in a resource-poor remote region of the country enabling access to timely diagnosis and care for hundreds of patients in a remote locality.

Subsequently, the same company reported a collaborative project with National Health System Resource Centre (NHSRC) set up under the National Rural Health Mission (NRHM) of the Government of India, where they explored the potential of teleradiology for improving healthcare access among community health centers, district, and sub-divisional hospitals in rural areas of Karnataka as an investigative project. They subsequently report their experience of implementing teleradiology service to a governmental network of 26 Community Health Centres (CHC), district (DH), and sub-district (SDH) hospitals of Tripura, a north-eastern state of India.^14^ These sites had analog X-ray equipment. Analog X-ray films were digitized using 8–10 Megapixel digital cameras. The JPEG images were converted to DICOM format using a web-based application and uploaded onto an in-house built cloud-based teleradiology workflow system (RADSpa). Images were transferred using a secure high-speed internet connection (4Mps and above) to the electronic workflow platform, which was distributed to the reporting radiologists. The electronic workflow platform had an integrated radiology information system (RIS) and picture archiving and communication system (PACS), which was used for reporting, archiving, record keeping, and billing. Between January 2018 and November 2021, 78,622 plain X-rays of a wide range of anatomical regions from head to toe were reported and these included both paediatric and elderly populations. The average turnaround time for priority reporting was around 3 h, and 8 h for routine examinations. They also report that 0.3% of all exams were marked as suboptimal in quality and thus could not be reported.^14^ These studies demonstrate the feasibility and methodology of establishing teleradiology setup in resource poor environments and emphasises the need for trained on-site technicians to support the teleradiology workflow, high speed internet connectivity and secure image transfer protocols. While the above studies give a sense of the volume of service delivery, there are no studies on the needs assessment for teleradiology services for rural population in India.

Indian college of Radiology and imaging (ICRI) released a white paper on teleradiology on behalf of Indian Society of Radiology and Imaging (IRIA) in 2021, which provides broad guidelines for the infrastructure, technical standards and secure transmission of imaging data for teleradiology practices in India.^25^ The white paper mentions the need for analog to DICOM conversion protocols and the use of technologies such as lossless compression, multithreaded or parallel transmission for achieving speedy image transfers. The document also suggests methods to achieve zero downtime which include redundancy for power supply, internet connectivity, networks, applications and database. In addition, the paper spells out who can practice teleradiology, clinical reporting and communication standards for teleradiology reports, and the need for peer review-based quality assurance measures.^25^ Recommendation on contractual agreement including statements on reimbursement policy pertaining the teleradiology service are also included.^25^

#### Artificial intelligence (AI)

At the time of data collection, there were numerous publications on AI. Due to the ongoing pandemic at the time, most of these studies were on chest X-ray diagnosis of COVID-19 infection. For the purposes of this review, we only included non-COVID-19 related AI studies (n = 11). These studies were mainly concordance studies on the development, validation and the testing phases of deep learning algorithms for diagnosis of specific disease entities on chest X-ray or CT scans.^26^, ^27^, ^28^, ^29^, ^30^, ^31^, ^32^, ^33^, ^34^, ^35^, ^36^ Typically, either radiology reports, specialist radiologist's interpretation or biopsy were the reference standards for these studies. Available evidence demonstrates feasibility, diagnostic accuracy and equivalence of AI assisted diagnosis with the image interpretation by experienced radiologist.^26^, ^27^, ^28^, ^29^, ^30^, ^31^, ^32^, ^33^, ^34^, ^35^, ^36^ AI tool for chest X-ray reporting is already being used in clinical practice for triaging of priority reporting by radiologists.^32^

AI tool called qTrack developed for chest X-rays by Qure AI company works on analog X-rays and can identify and localize 29 different abnormalities on chest X-rays. This makes it well-suited for tuberculosis screening in remote areas. Currently, five districts (Chikaballapur, Koppal, Bellary, Belgaum, and Bagalkot) in Karnataka have been chosen for pilot implementation of Screening for Tuberculosis using Artificial intelligence in Remote Radiology (STAR) facilities.^37^ STAR is a PPP between the State TB Office, the Association of Public Health Technologists (APT), and the Karnataka Health Promotion Trust (KHPT) funded by India Health Fund and supported by Qure AI.^37^ In this program, the peripheral health worker captures a digital photograph of the analog X-ray mounted on a view box using a smartphone and then uploads to the qTrack app from the photo gallery, and the AI platform would instantly analyze the X-ray. The expected benefit of the program is a decrease in the time taken for diagnosis and improved quality of interpretation by minimizing the interobserver variability. Initial reports since the start of the STAR program in June 2021 state that over 6300 chest X-rays have been subjected to AI-based analysis. Out of them, 1625 chest X-rays were picked up by the AI algorithm as suggestive of tuberculosis. A positivity rate of 20% was found among those who were subsequently subjected to a Cartridge-Based Nucleic Acid Amplification Test (CBNAAT).^37^

Another recognized value of AI is in reporting critical findings such as an intracranial bleed on brain CT scans and in mammography interpretation for breast cancer detection.^26^^,^^33^ Thus, AI has shown the potential in improving access to health and can to some extent address radiologists’ shortages. However, there is no literature currently on the cost-effectiveness, evaluation, and implementation of AI for image interpretation.

#### Data security

Data security is usually thought of as a triad of Confidentiality, Integrity, and Availability (CIA). Research and developments in data security are central to the development and expansions of teleradiology, Picture Archiving, and Communication Systems (PACS) and e-Health services within the country. So, we assessed the studies that reported technologies that enabled data security in the teleradiology domain. One of the earlier studies reports sFTP for safe image transfer between the client and the teleradiology service provider. sFTP utilizes Secure-Shell encryption (SSH) for better transmission security.^24^ Newer studies report other methods of encryption such as an enhanced chaos-based scrambling approach for DICOM images. In this approach, the Pseudo-Random Number Generators (PRNGs) such as the Linear Congruential Generator (LCG) and XOR Shift Generator (XSG) are combined with a logistic 2D coupled chaotic map to achieve better encryption of DICOM images, which in turn enable better protection of patient confidentiality while using RIS for storage and image transfer.^38^ A study assessing data integrity during imaging transfer reports secure transmission of the entire dataset using a method called Advanced Encryption Standard (AES).^39^ Another study reports a novel method of DICOM image encryption of any pixel depth using JSMP map and was reported to be promising for sharing sensitive health information.^40^ The importance of data protection and security using an IPsec VPN tunnel between the hospital network and the teleradiology providers, and DICOM image encryption using the DICOM TLS protocol has been emphasized in the recent ICRI white paper on teleradiology.^25^ The available literature shows the methods available for secure image transfers. However, there is no published literature on the extent to which these methods are put to practice by the various teleradiology facilities across India. Similarly, there is no published literature on the strengths and challenges faced in the efforts to ensure data security.

#### Physicians—local radiologists, local physicians, and teleradiologists

At the time of this review, there were only around 20,500 radiologists registered with the Indian Society of Radiology and Imaging.^41^^,^^42^ This translates to only 1 per 100,000 population compared to 1 per 10,000 in the United States. Also, there has been a quantum leap in the radiology workload with radiology taking the center stage in the diagnostic and management pathways. However, the number of faculty positions for radiologists and MD Radiology degree seats have not increased in proportion to the increase in the radiology workload over the years or the needs of India. Also, subspecialty radiologists are scarce and are mainly concentrated in large tertiary care hospitals and academic institutions. These factors have contributed to the acute shortages of radiologists in our country.^21^ Radiologists employed in large numbers by teleradiology companies may have contributed to the shortages of radiologists. Adding to this problem is the trend of Indian radiology post-graduates and senior residents writing multiple foreign exams.

Though teleradiology has several advantages, radiology jobs are increasingly being likened to the cybercafé with multiple cubicles of the café being replaced by high-resolution workstations. Teleradiologists increasingly work in isolated corners, sometimes basements or their homes. There are high pressures to work faster to stick to prescribed turn-around times with accuracy. These factors compounded by radiology services seen as profit-making ventures by administrators and investors have resulted in a decline in radiologists’ interactions with patients and other clinical colleagues. This has resulted in the loss of enthusiasm, decreased work satisfaction, decreased professional relationships, decline in support, and a “feeling of isolation”.^21^ These factors combined with fear of AI replacing radiologists may cause a decline in the number of takers for the specialty in the future.

The high investment needed for starting radiology services would need of good workload at a competitive price to obtain returns on the investment. This translates to fewer radiology facilities in the periphery, high cost and the need for travelling long distances to access advanced diagnostics. This also meant that the radiologists working in the periphery were only able to practice a part of their skillset. These factors along with the attractive job offers at an early stage of career by large hospitals and teleradiology companies have contributed to the concentration of radiologists in large cities. However, with improved high speed internet connectivity and the rapidly expanding teleradiology milieu in India, Indian radiologists may slowly move to the periphery where they can deliver to the local needs by practicing X-rays and ultrasounds, and at the same time maintain their skillsets by reporting advanced imaging such CT and MRI scans on teleradiology platforms in their free time.

#### Operational aspects and challenges

The challenges perceived for widespread adaptation of teleradiology and technology solutions to improve access include: understaffing of government hospitals; lack of in-house radiology expertise to supervise the service; quality issues such as poor quality images, and incomplete studies, which in turn may be due to training gaps or equipment related; lack of service engineers for equipment maintenance in the peripheral hospitals; lack of clinical information and feedback system to address issues may affect the quality of teleradiology reports; travel time, poor connectivity to the facility and the cost of service can deter the usage of technology solutions; power outages and lack of high-speed internet connectivity; and lastly social beliefs, resistance to modern diagnostic facilities, resistance to technology, and change, in general, are also reported as challenges.^14^^,^^20^^,^^21^ In addition to these, there are challenges to governance and regulatory issues that surround teleradiology practices.^23^ There are also unaddressed issues such as reimbursement of services utilizing teleradiology by medical insurance companies and medico-legal responsibilities of teleradiologists and teleradiology providers.^21^ There were also no studies on the needs assessment, cost-effectiveness, and evaluation of teleradiology solutions, artificial intelligence, or other technology solutions at the primary health care level.

A study titled “We sold the buffalo to pay for a brain scan’—a qualitative study of rural experiences with private mental healthcare providers in Uttar Pradesh, India” highlighted the disconnect between the needs at the grass root level and the existing referral pathways and the cost for diagnosis and treatment. This study also highlighted the need for regulation of private and public providers and the need for better utilization of health resources.^43^ Though this work was on mental health, it highlights the out-of-pocket expenditure incurred by patients in rural areas for diagnosis and its effects on them and their families.

### Public-private partnerships (PPP) and shared interoperability

PPP was adopted as an important supplementary strategy by the National rural health mission (NRHM) in the 11th five-year plan (2007–2012), to improve healthcare delivery, especially primary healthcare in states that had weak health indicators and health infrastructure.^11^ PPP was proposed as potential solution in two areas–one for radiology services and the other for improving access to healthcare. Broad regulatory and partnership frameworks for practical and cost-effective PPP models must be based on “value for money” and “clearly defined sharing of risks”.^11^ PPP was sort to address the absence of or poor-quality radiology equipment in the public health delivery systems; secondly, to rapidly expand the number of mobile health vans equipped with basic diagnostic facilities (X-ray, ultrasound), lab tests and a mini-clinic as a means of providing access to primary healthcare services to difficult to reach terrains with poor connectivity; and thirdly to address the issue of shortfall in health expertise.

#### Trial and implementation of PPP models

For the first time, private services were contracted for government hospital installations and the running of radio-diagnostic equipment such as X-ray machines and CT scanners in seven government hospitals in West Bengal. This strategy was seen as a means of providing cost-effective, round-the-clock (24 × 7) diagnostic services to public hospitals with limited resources, and was hoped to bring health expertise to public hospitals to provide a better quality of care.^11^ Under the National Health Mission (NHM), Andhra Pradesh was the first state to roll out a hybrid model of free-of-cost radiology and laboratory service in 2016 through a PPP model across its district hospitals and PHCs and was called the NTR Vaidya Pariksha scheme.^44^

Following this report, three types of PPP initiatives were started in several states to address the issue of access to better diagnostics and these included the ‘Hub and Spoke’ model, outsourcing of diagnostics services, and contracting-in.^44^

In the outsourcing model, high technology, high cost, and lower frequency diagnostic services were outsourced to the private service providers while low-cost, high-volume tests and those not requiring highly skilled manpower were to be undertaken within public health facilities. Services of specialists, such as radiologists, pathologists, microbiologists, etc, were contracting-in when in-house expertise was not available. FDI-lab implementation report dated 31st August 2018, states that such facilities have been implemented in 31 States/UTs. PPP mode was reported in 9 States and the in-house mode was reported in 22 States/UTs.^44^

PPP that surrounds the teleradiology initiative is driven by the shortages of specialist clinicians, especially radiologists. Teleradiology is a cost-effective and viable solution through which digitized images of X-rays and other radiology investigations like ultrasound, CT, and MRI are transmitted to the service provider, and the reports are received within a stipulated time frame. FDI-teleradiology implementation status report as of 31st August 2018 states the PPP mode of teleradiology services in nine States, CT scan installations in 13 states with the in-house mode of implementation in 11 states/UTs.^44^ Few select examples of activities operating in PPP mode have been described in the relevant sections above.

#### Evaluation of PPP models

There are no evaluation studies on the mobile medical unit that function in PPP mode. However, two preliminary studies evaluate the initial results of mobile digital X-ray units in the tuberculosis control program and demonstrate the feasibility of such a program in a PPP model.^7^^,^^45^ There are very few evaluation studies on the PPP models for Radiology services. One of them brings out the challenges of radiology services functioning in PPP mode in Bihar.^46^ These challenges included the weak public sector, weak organizational capacity, poor monitoring of the quality of service, weak regulations, and failure of establishing a working relationship between local hospital administrators and centralizing contractors.^46^ Another study evaluating the quality of mammograms obtained using mobile units of RAD-AID Asha Jyoti Mammogram program highlighted the training gaps among the mammography technicians and the challenges in maintaining quality despite training.^12^ In 2018, a WHO-directed evaluation of the NTR Vaidya Pariksha program was conducted. The results of the evaluation showed that 52% of tests were performed at the PHC level and 6% in district hospitals (DH). This is probably a result of improved access at the primary care level decreasing the need for traveling to the DH to access basic diagnostic tests. The evaluation reported a 55% reduction in out-of-pocket expenditure on diagnostics.^44^ This example showed the value of successful delivery of imaging care and diagnostics in the PPP model in improving access to health and decreasing costs at primary health care. The report also listed the key enablers for the success of the program (Box 1 in supplementary file 2).^44^

Table 4 summarises the materials available on each outcome of interest. The key findings that emerged from the review are as follows:

▪ The boom of the teleradiology industry in India is not adequately represented in the available literature and there is insufficient data on the scale of teleradiology networks in the country. Limited available literature has mainly emerged from select large-volume teleradiology providers and subject matter experts.
▪ Data available so far demonstrates the feasibility of using teleradiology to improve access to diagnostics in remote and resource-poor geographical areas. Also, bring out the strengths and challenges of using teleradiology services.
▪ Numerous concordance studies on AI algorithms show results comparable to a standard radiologist for the interpretation of common pathologies on chest radiographs and for diagnosing intracranial bleeds on the brain CT scans.
▪ Preliminary results of the use of artificial intelligence and mobile van-mounted digital X-ray units for tuberculosis control in PPP models are promising and have immense potential for addressing health worker shortages and improving access to difficult-to-reach terrains of India.
▪ PPP models have been widely adopted across the country for high-resource low-frequency radiology services. Available literature demonstrates the strengths and weaknesses of these models.
▪ Both teleradiology services and artificial intelligence-based services appear to have adapted to the still widely used analog X-ray units across the country by adopting methods of digitizing the analog X-ray films.
▪ Alternative image display units such as iPad, mobile phones, and social media platforms such as WhatsApp may be adequate at specific points of care such as dental services, trauma, and emergency.
▪ The literature available on data security and data protection is sparse. Two examples of teleradiology service to the north-eastern states briefly mention secure transmission of patient information and imaging data.
▪ There are knowledge gaps regarding the expected standards, laws, and regulations governing data protection, patient confidentiality, and quality standards of teleradiology practices. Similarly, there are no guidelines and regulations that surround reimbursements from teleradiology services providers and laws around medical litigations in teleradiology practices.
▪ There is no data on the evaluation and cost-effectiveness of teleradiology and other newer technologies in improving access to primary healthcare.
▪ There are data gaps in the needs assessment; facilitators and barriers to the implementation of teleradiology and newer technologies in primary healthcare settings.

**Table 4:** Studies that provided data on the key moments in the imaging care of the rural population.

Technologies enabling connectivity and access	Mobile digital X-ray Units^7^, ^8^, ^9^, ^10^, ^11^, ^12^, ^13^, ^14^ Conversion of analog to digital X-rays^15^^,^^16^ Image display^10^^,^^17^^,^^18^ Cloud/web-based Image transfer/DICOM viewers^19^, ^20^, ^21^ Teleradiology ^16^^,^^22^, ^23^, ^24^, ^25^, ^26^, ^27^ Artificial intelligence^28^, ^29^, ^30^, ^31^, ^32^, ^33^, ^34^, ^35^, ^36^, ^37^, ^38^, ^39^
Data security	^26^^,^^27^^,^^40^, ^41^, ^42^
Physicians	^23^^,^^47^
Cost	^12^^,^^13^^,^^44^
Operational challenges	^16^^,^^22^^,^^23^^,^^25^^,^^43^
Shared interoperability	^9^^,^^11^^,^^14^^,^^16^^,^^21^^,^^26^^,^^39^^,^^44^, ^45^, ^46^

## Discussion

Given that there are only around 20,500 radiologists in our country to serve a population of 1.4 billion,^42^ the need for increasing the reach and capacity of our existing radiologists is paramount. Teleradiology and newer technologies including AI are enablers to achieve this. The examples available in the literature and the findings of this review support this notion.

Teleradiology in India is essentially carried out by private actors with a very large activity. But there is too little published literature to assess this activity in terms of its reach, effectiveness, and its cost, and there is insufficient published literature on the various components of the teleradiology practice. These aspects are important knowledge gaps that do not allow us to have an exact global vision of teleradiology in India. Large Indian teleradiology companies have been delivering services to other countries for over two decades now. While this is a contributing factor to the shortages of radiologists available for the needs in India, it has been instrumental in understanding and bringing home the infrastructure and the means of setting up good teleradiology services within India. The rich experience gained has partly translated into the teleradiology boom and the thriving teleradiology practice in India in the current times.

Though there is no published literature, in the author's opinion the activity within the country seems more than the activity provided for other countries. While the competitive prices offered by Indian teleradiology services had helped it stay afloat in the overseas market for this long, especially for night-time emergency services, the shortages of radiologists in general and that of English-speaking board-certified radiologists, in particular, is a major limiting factor for the expansion of its global reach.^47^ Thus, even large Indian teleradiology companies are becoming increasingly reliant on the growing market appetite for teleradiology services within India. There is an increasing trend of both small and large hospitals in India moving towards outsourcing their radiology services to teleradiology companies. This is a reflection of the shortages in radiologists, and the difficulty in employing and retaining radiologists at a competitive price when compared to teleradiology companies, which employ them in large numbers like the IT sector of India. The few radiologists in smaller cities and semi-urban areas mostly deliver X-ray and ultrasound services locally. There is an increase in the number of CT and MRI scanners that are owned and managed by non-radiologists who partner with local physicians for their clientele and outsource reporting to teleradiology companies. This is partly because the small scan centres cannot afford to employ a full-time radiologist.

There is a need to maintain a healthy balance between the services delivering to the needs of India and those delivering to other countries. Keeping a share of overseas clientele would help in imbibing quality standards, bring home additional revenue which can partly support the cost for local services, and allow fresh ideas to flow into our system. A global network of radiologists in a teleradiology framework has transformed trauma and emergency care because of their capacity for providing round-the-clock service. However, we are far from making similar services available even in large cities of India and the idea is even more far-fetched when it comes to primary care gaining access to round-the-clock services.

Addressing the challenges of technology solutions, especially those related to quality and cost issues will mainly drive their widespread implementation at the primary healthcare level. The quality needs of teleradiology are closely linked with the training needs of technicians who can effectively participate in the teleradiology workflow. This is an important aspect of the success of teleradiology at the periphery. The sheer volume of service that is needed would require the technicians to be able to participate in activities beyond optimal image acquisition and digitization of images. They might have to be trained to bridge the gaps in the availability of service engineers to perform small troubleshooting tasks in both the equipment and the teleradiology workflow.

While minimum standards are ensured by large teleradiology services where there are quality checks and a degree of second read for subspecialty reporting, it is not a widespread practice. The range of market prices for teleradiology services and the variable quality of the service in India is a direct reflection of this fact. In the current teleradiology practice environment, only a few services provide means for the end user to interact with the reporting teleradiologist and the majority have adopted a ‘cybercafé’ approach with little scope for feedback and continuous improvement on the job. There is an urgent need for guidelines for minimum standards, safety nets including limits on the number of reports per day per radiologist, and a professional framework for teleradiology practices. It will be important to take care of these aspects before attempting to standardize the cost of teleradiology services.

We are seeing an increasing trend among Indian Radiology residents and senior residents to get overseas degrees and there are numerous commercial preparatory courses for these exams across the country. The contributing factors for these trends are examination centers located in India, lucrative overseas job opportunities with better working conditions, opportunities for short-term overseas fellowships, better job opportunities in Indian teleradiology companies for candidates who have additional overseas degrees, and opportunities to work for overseas teleradiology companies on their return to India. These trends may also add to the shortage of Indian radiologists. The positive impact of this trend may be improved quality of Radiologists, more subspecialty expertise, and redesigning of the Indian Radiology curriculum and examination systems to make it at par with other overseas examinations. The negative impacts could range from a delay in the radiologists joining the workforce to a degree of facilitated brain drain.

The true paradigm shifts in healthcare delivery going forward will be the integration of artificial intelligence (AI) algorithms into the diagnostic workflow. Such deployment of AI-based diagnostic algorithms into teleradiology workflow will have the greatest effect in addressing shortages of radiologists and in optimizing the available workforce. Though literature from primary healthcare settings is sparse, the available evidence supports the value addition that teleradiology and newer technologies including AI will bring to emergency and trauma care, to improve access to imaging care and subspecialty care to remote geographical areas, and support and enable population-based screening programs for tuberculosis and cancer. At present, AI is being used extensively within scanners to optimize image quality and decrease scan time. While AI is a compliment rather than a replacement for radiologists in most areas, AI can be effectively used as a first read to differentiate normal from abnormal chest X-rays; identify certain specific diseases such as pneumonia, pleural effusion, TB, intracranial bleed, fractures, and breast cancer with a high degree of accuracy. The most common imaging services that would be needed at primary care are chest X-rays, obstetric ultrasound, plain CT brain, and mammography, and AI can deliver to some of these needs. AI can potentially be used as a triage to identify imaging that needs an urgent second read by radiologists located remotely. There are however knowledge gaps in the needs assessment, required quality standards, cost, facilitators, and barriers for implementation, and the evaluation of AI in a primary healthcare setting.

The regulations that surround personal information and digital data protection in India are still evolving. Data protection and secure image transfer is a complex issues, and we need guidelines on the minimum standards for the tools used to achieve this in teleradiology and telehealth practices. Most recently on November 18, 2022, the Digital Data Protect Bill 2022 was released for public consideration by the Union Ministry of Electronics and Information Technology.^48^ The new bill proposes a comprehensive personal data protection regime but eases the restrictions on non-personal data collection and transfer of data across borders. The bill also levies heavy fines for non-compliance indicating private organizations to formulate stringent data protection programs. To comply with the new bill, the radiology businesses leveraging on AI, deep learning and teleradiology must maintain a defensive inventory of data, have stringent management of requests for data access, manage third-party-related risks of personal data breaches, and minimize data retention to the least amount that is required.

For teleradiology and other technologies to aid in improving access to primary health care, the critical step is to create a robust digital infrastructure. The National Digital Health Mission (NDHM) 2020 envisions creating a national digital health ecosystem, which supports universal health coverage across India.^49^ Findings of our review will be of use in shaping the plan, the design, and the execution of the NDHM.

A review of both white and grey literature is one of the biggest strengths of this review. Choosing to adapt search strategies to each database and reviewing grey literature published by the government, teleradiology service providers, and other relevant stakeholders helped us gather materials about the knowledge gaps that surfaced from the white literature review alone.

However, our review is limited by the search of white literature performed only till March 2022. Since radiology research in newer technologies especially AI is rapidly evolving, publications that followed March 2022, may not have been included in this review. We restricted the search to the English language. We may have missed materials published in other Indian languages which might have had information relevant to this review. The reach of teleradiology networks could have been mapped using information available on social media platforms. However, undertaking such a task was beyond the scope of the current review.

In conclusion, the available evidence suggests that teleradiology, particularly when combined with artificial intelligence-based algorithms and mobile digital imaging units can address shortages of radiologists; improve access to health; strengthen programs aimed at population screening for the detection and early diagnosis; and emergency care. Alongside the large-scale implementation of teleradiology services for primary healthcare, there is an urgent need to address shortages of radiologists and technicians who can support the service. However, there is insufficient data on the scale of teleradiology activity and networks within India. There is no data on the regulations governing quality standards, data protection, patient confidentiality, and the cost of teleradiology services. There is insufficient data on the needs assessment; cost-effectiveness; the facilitators and barriers for implementation of teleradiology and newer technologies in primary healthcare settings; and there is no data on the direct impact this will have on individual health outcomes.

## Contributors

AJ: conceptualisation, methodology, resources, software, writing - review & editing.

SG: conceptualization, methodology, resources, software, writing - review & editing.

AK: conceptualisation, methodology, resources, software, writing - review & editing.

AC, VK, AA, MK: Directly accessed and verified the underlying data in all research articles.

AC, VK, AA, MK, HT, and TD were the authors from the academic team.

All authors were responsible for the decision to submit the article.

## Declaration of interests

AC, VK, AA, MK, HT, TD, SG: We declare no competing interests.

AJ: Discloses holding multiple patents in domains of deep learning with applications in Radiology, natural language processing, disease-specific algorithms for interpreting Radiology images, and ESOPs grant for contributions to Qure.ai.

AK: Discloses his position as a CEO and Chief Radiologist of Teleradiology Solutions, Bengaluru; and the president of Society of Emergency Radiology.

## Appendix A Supplementary data

Enablers for the successful delivery of imaging care in the PPP model

Abstract translated into Hindi

Abstract translated into Tamil

Abstract translated to English
