# Supplementary material for: Teleradiology and technology innovations in radiology: status in India and its role in increasing access to primary health care
Source: Lancet Reg Health Southeast Asia. 2023 Apr 14;23:100195. doi: 10.1016/j.lansea.2023.100195 (PMC10884973; doi:10.1016/j.lansea.2023.100195)
Supplement: Enablers for the successful delivery of imaging care in the PPP model [file mmc1.docx]

**Search strategy:**

The search strategy was based on the Preferred Reporting Items for Systematic Reviews and Meta-Analyses (PRISMA) 2020 flowchart for systematic reviews. PubMed, Google Scholar, IndMed, Cochrane database were used as the search engine. The data was obtained from studies via databases and registers. Various blogs, news articles and other sources of grey literature were also included. The following combinations of keywords were searched for: ” ("Zero footprint"[Title/Abstract]) AND (India*[Title/Abstract]) - Schema: all, ("Zero footprint"[Title/Abstract]) AND (India*[Title/Abstract]), ((radiolog*[Title/Abstract]) AND (apps[Title/Abstract])) AND (India*[Title/Abstract]), (("medical imaging"[Title/Abstract]) AND (informatics[Title/Abstract])) AND (India*[Title/Abstract]) - Schema: all, (("medical imaging"[Title/Abstract]) AND (informatics[Title/Abstract])) AND (India*[Title/Abstract]), ((radiolog*[Title/Abstract])) AND (informatics[Title/Abstract])) AND (India*[Title/Abstract]), ("cloud computing"[Title/Abstract]) AND (India*[Title/Abstract]), ((cloud[Title/Abstract]) AND (radiolog*[Title/Abstract])) AND (India[Title/Abstract]), (("point of care"[Title/Abstract]) AND (radiolog*[Title/Abstract])) AND (India*[Title/Abstract]), Screening[Title/Abstract] AND radiolog*[Title/Abstract] AND India[Title/Abstract], (mobile[Title/Abstract]) AND (CT[Title/Abstract] OR MRI[Title/Abstract] OR mammogra*[Title/Abstract] OR X-ray*[Title/Abstract] AND India*[Title/Abstract]), Mobil*[Title/Abstract] AND ultrasound[Title/Abstract] AND India*[Title/Abstract], "smart phone"[Title/Abstract] OR whatsapp[Title/Abstract] OR instagram[Title/Abstract] OR twitter[Title/Abstract] OR "social media"[Title/Abstract] AND radiolog*[Title/Abstract] AND India*[Title/Abstract], whatsapp[Title/Abstract] OR instagram[Title/Abstract] OR twitter[Title/Abstract] AND radiolog*[Title/Abstract], "Smartphone*"OR whatsapp[Title/Abstract] OR instagram[Title/Abstract] OR twitter[Title/Abstract] AND radiolog*[Title/Abstract], Teleradio*[Title/Abstract] AND India*[Title/Abstract], "Artificial intelligence" OR AI OR "deep learning" AND radiolog* AND India, Screening[Title/Abstract] AND radiolog*[Title/Abstract] OR Imagi*[Title/Abstract] AND India[Title/Abstract]”.

A total of 2503 of articles were obtained. These articles were screened by title for relevance by two independent authors and 156 articles were selected for full-text screening. Of these 156, a total of 84 potentially eligible publications required the full­ text version for further investigation. After a review of the full-text version, a total of 41 reports were excluded either because they were not relevant to this review (n=34) or because these studies that were not from India (n=7). The remaining 43 publications fulfilled the inclusion criteria. Of the 43 included, there were n=19 cross-sectional studies, that included prospective (n=10) and retrospective (n=9) studies. Other studies included qualitative studies (n=3), and editorial pieces or commentaries (n=5). The study design was not mentioned in some studies (n=16) included. We used the Critical Appraisal Skills Programme (CASP) checklist to evaluate the quality of the studies. The quality of studies selected for review were heterogeneous with the majority being average to low in quality. A total of 52 articles that can be categorized as grey literature and fulfilled the inclusion criteria were considered for analysis. These included blogs (n=11); news articles (n=27); conference abstracts (n=10); policies and articles published by the government (n=2) and government schemes (n=2). A summary of the selection process is presented in Figure 1.
