# Supplementary material for: Teleradiology and technology innovations in radiology: status in India and its role in increasing access to primary health care
Source: Lancet Reg Health Southeast Asia. 2023 Apr 14;23:100195. doi: 10.1016/j.lansea.2023.100195 (PMC10884973; doi:10.1016/j.lansea.2023.100195)
Supplement: Abstract translated into Hindi [file mmc2.docx]

Box 1: Enablers for the successful delivery of imaging care in the PPP model (from 2018 WHO evaluation). (*Reference number 44*)

1. Leadership
2. Allocation of adequate budget.
3. High political and administrative commitment
4. Phased rollout
5. Availability of tests in the designated facilities
6. Overcoming initial resistance in prescribing the tests to the service provider and trust building between the doctors and the service providers
7. Timely payments to the service providers
8. Penalties to the service providers for non-delivery of service
9. Framework for monitoring service deliver and its quality.
10. Intensive campaign to inform the community about the program.
