# Supplementary material for: Teleradiology and technology innovations in radiology: status in India and its role in increasing access to primary health care
Source: Lancet Reg Health Southeast Asia. 2023 Apr 14;23:100195. doi: 10.1016/j.lansea.2023.100195 (PMC10884973; doi:10.1016/j.lansea.2023.100195)
Supplement: Abstract translated into Tamil [file mmc3.docx]

**Hindi translation of the abstract**

**शीर्षक:** रेडियोलॉजी में टेलीरेडियोलॉजी और प्रौद्योगिकी नवाचार: भारत में स्थिति और प्राथमिक स्वास्थ्य देखभाल तक पहुंच बढ़ाने में इसकी भूमिका।

**अमूर्त:**

**पृष्ठभूमि:** देश में रेडियोलॉजी सुविधाओं का असमान वितरण है। हालांकि, कृत्रिम बुद्धिमत्ता (एआई) से जुड़े टेलीरेडियोलॉजी और नवाचारों में मौजूदा स्वास्थ्य प्रणालियों को मजबूत करने और प्राथमिक स्वास्थ्य देखभाल तक पहुंच में सुधार करने की क्षमता है।

**उद्देश्य:** भारत में एआई सहित टेलीरेडियोलॉजी, और मेडिकल इमेजिंग इंफॉर्मेटिक्स की स्थिति पर उपलब्ध साहित्य को समेकित करना, और स्वास्थ्य तक पहुंच में सुधार, स्वास्थ्य देखभाल प्रणालियों की दक्षता और घटती लागत में इसकी भूमिका।

**डेटा स्रोत:** PubMed, Google विद्वान, IndMed, Cochrane डेटाबेस, और सेवा प्रदाताओं की रिपोर्ट सहित गैर-शैक्षणिक साहित्य; सरकारी दस्तावेज; राय टुकड़े, और विषय विशेषज्ञों की समीक्षा।

**खोज मानदंड और योग्यता मानदंड:** विषय की प्रकृति के कारण और जितना संभव हो उतना समावेशी होने के कारण, व्यापक शब्दों का उपयोग करके एक खोज की गई, जो चिकित्सा इमेजिंग उपकरणों या सूचना विज्ञान के उपप्रकारों के लिए गैर-विशिष्ट थी। मुख्य रूप से भारत में संचालित कार्य, 2005 से मार्च 2022 के बीच अंग्रेजी भाषा में प्रकाशित, और पूर्ण पांडुलिपियों वाले शामिल थे।

अध्ययन मूल्यांकन और संश्लेषण के तरीके: दो लेखकों ने स्वतंत्र रूप से पूर्ण-पाठ समीक्षा के लिए समावेशन मानदंड के खिलाफ सार की जांच की और एक वरिष्ठ लेखक ने विसंगतियों का समाधान किया। एक समीक्षक द्वारा डिस्टिलरएसआर सॉफ्टवेयर का उपयोग करके डेटा निकाला गया और दूसरे द्वारा क्रॉस-चेक किया गया। क्रिटिकल अप्रेज़ल स्किल्स प्रोग्राम (CASP) चेकलिस्ट का उपयोग करके बायस का मूल्यांकन किया गया था।

**परिणाम:** 43 मूल लेख और 52 गैर-शैक्षणिक सामग्रियों की अंत में समीक्षा की गई। मूल लेखों में व्यापक रुझान थे: टेलीरेडियोलॉजी (एन = 7), मोबाइल डिजिटल इमेजिंग इकाइयों (एन = 9), कृत्रिम बुद्धि (एन = 16) का उपयोग कर कनेक्टिविटी (एन = 17); मोबाइल डिवाइस और स्मार्टफोन एप्लिकेशन (एन = 7); डेटा सुरक्षा (n=7) और वेब-आधारित तकनीक (n=2); सार्वजनिक-निजी भागीदारी के साथ साझा अंतरसंचालनीयता (n=9); लागत (एन = 2); सामंजस्य (एन = 19); आकलन की जरूरत है (एन = 12); मूल्यांकन (एन = 4); कार्यान्वयन (एन = 2)। समीक्षा में 10 भारतीय राज्यों के डेटा शामिल थे जिनमें से कुछ ग्रामीण सेटिंग्स (एन = 9) से थे। टेलीरेडियोलॉजी और एआई की भूमिका निमोनिया, तपेदिक, इंट्राक्रानियल ब्लीड और आपातकालीन रेडियोलॉजी में सबसे अधिक प्रदर्शित हुई।

**मुख्य निष्कर्षों के निष्कर्ष और निहितार्थ:** उपलब्ध साक्ष्य बताते हैं कि टेलीरेडियोलॉजी, विशेष रूप से एआई और मोबाइल डिजिटल इमेजिंग इकाइयों के साथ मिलकर रेडियोलॉजिस्ट की कमी को दूर कर सकती है; पहचान और शीघ्र निदान के लिए जनसंख्या जांच के उद्देश्य से कार्यक्रमों को मजबूत करना; और आपातकालीन देखभाल। हालांकि, भारत के भीतर टेलीरेडियोलॉजी नेटवर्क के पैमाने पर पर्याप्त डेटा नहीं है; गुणवत्ता मानकों, डेटा संरक्षण और गोपनीयता को नियंत्रित करने वाले नियम; आकलन की आवश्यकता है; लागत; प्राथमिक स्वास्थ्य देखभाल सेटिंग्स में टेलीरेडियोलॉजी और नई तकनीकों के कार्यान्वयन के लिए सुविधा और बाधाएं; और इसका सीधा प्रभाव व्यक्तिगत स्वास्थ्य परिणामों पर पड़ेगा।

**कुंजी शब्द:** टेलीरेडियोलॉजी; मोबाइल-टेलीरेडियोलॉजी; प्राथमिक स्वास्थ्य देखभाल; पहुँच; तकनीकी; कृत्रिम होशियारी; सार्वभौमिक स्वास्थ्य कवरेज

**मुख्य निष्कर्ष / हाइलाइट्स:**

1. उपलब्ध डेटा दूरस्थ भौगोलिक क्षेत्रों में इमेजिंग देखभाल तक पहुंच में सुधार के लिए टेलीरेडियोलॉजी को लागू करने की व्यवहार्यता, ताकत और चुनौतियों को प्रदर्शित करता है।
2. एआई एल्गोरिदम पर समनुरूपता अध्ययन ऐसे परिणाम दिखाते हैं जो छाती के रेडियोग्राफ़ पर सामान्य विकृति की व्याख्या के लिए रेडियोलॉजिस्ट की तुलना में हैं और मस्तिष्क सीटी स्कैन पर इंट्राक्रैनील रक्तस्राव के निदान के लिए हैं।
3. सार्वजनिक-निजी भागीदारी (पीपीपी) मॉडल में तपेदिक नियंत्रण के लिए कृत्रिम बुद्धिमत्ता और मोबाइल डिजिटल एक्स-रे इकाइयों के उपयोग के संबंध में प्रारंभिक परिणाम आशाजनक हैं और इनमें स्वास्थ्य कर्मियों की कमी को दूर करने और पहुंच में सुधार करने की अपार संभावनाएं हैं।
4. उच्च-संसाधन कम-आवृत्ति रेडियोलॉजी सेवाओं के लिए देश भर में पीपीपी मॉडल व्यापक रूप से अपनाए गए हैं। उपलब्ध साहित्य इन मॉडलों की ताकत और कमजोरियों को प्रदर्शित करता है।
5. दोनों टेलीरेडियोलॉजी और आर्टिफिशियल इंटेलिजेंस-आधारित सेवाओं ने एक्स-रे फिल्मों को डिजिटाइज़ करने के तरीकों को अपनाकर देश भर में व्यापक रूप से उपयोग की जाने वाली एनालॉग एक्स-रे इकाइयों को शामिल करने के लिए अनुकूलित किया है।
6. वैकल्पिक छवि प्रदर्शन इकाइयां जैसे आईपैड, मोबाइल फोन और व्हाट्सएप जैसे सोशल मीडिया प्लेटफॉर्म कुछ विशिष्ट नैदानिक परिदृश्यों जैसे दंत चिकित्सा सेवाओं, विशिष्ट बीमारियों के एक्स-रे, आघात और इंट्राक्रैनियल रक्तस्राव में पर्याप्त हैं।
7. डेटा सुरक्षा और डेटा सुरक्षा पर साहित्य विरल है।
8. डेटा सुरक्षा, रोगी गोपनीयता, और टेलीरेडियोलॉजी प्रथाओं के गुणवत्ता मानकों को नियंत्रित करने वाले विनियम स्पष्ट नहीं हैं।
9. मूल्यांकन, लागत-प्रभावशीलता, आवश्यकता मूल्यांकन पर ज्ञान अंतराल हैं; प्राथमिक स्वास्थ्य सेवा तक पहुंच में सुधार के लिए टेलीरेडियोलॉजी और अन्य नई तकनीकों का उपयोग करने के कार्यान्वयन में सुगमकर्ता और बाधाएं।
