# Supplementary material for: Teleradiology and technology innovations in radiology: status in India and its role in increasing access to primary health care
Source: Lancet Reg Health Southeast Asia. 2023 Apr 14;23:100195. doi: 10.1016/j.lansea.2023.100195 (PMC10884973; doi:10.1016/j.lansea.2023.100195)
Supplement: Abstract translated to English [file mmc4.docx]

**Tamil translation of the abstract**

**தலைப்பு:** கதிரியக்கத்தில் டெலிரேடியாலஜி மற்றும் தொழில்நுட்ப கண்டுபிடிப்புகள்: இந்தியாவின் நிலை மற்றும் ஆரம்ப சுகாதார சேவைக்கான அணுகலை அதிகரிப்பதில் அதன் பங்கு.

**சுருக்கம்:**

**பின்னணி:** நாட்டில் கதிரியக்க வசதிகள் சமமற்ற முறையில் விநியோகிக்கப்படுகின்றன. இருப்பினும், டெலிரேடியாலஜி மற்றும் செயற்கை நுண்ணறிவு (AI) சம்பந்தப்பட்ட கண்டுபிடிப்புகள் ஏற்கனவே உள்ள சுகாதார அமைப்புகளை வலுப்படுத்தவும், ஆரம்ப சுகாதார பராமரிப்புக்கான அணுகலை மேம்படுத்தவும் திறனைக் கொண்டுள்ளன.

**குறிக்கோள்:** டெலிரேடியாலஜியின் நிலை மற்றும் இந்தியாவில் AI உள்ளிட்ட மருத்துவ இமேஜிங் இன்ஃபர்மேடிக்ஸ் மற்றும் ஆரோக்கியத்திற்கான அணுகலை மேம்படுத்துவதில் அதன் பங்கு, சுகாதார அமைப்புகளின் செயல்திறன் மற்றும் செலவைக் குறைப்பதில் கிடைக்கக்கூடிய இலக்கியங்களை ஒருங்கிணைத்தல்.

**தரவு ஆதாரங்கள்**: PubMed, Google Scholar, IndMed, Cochrane தரவுத்தளம் மற்றும் சேவை வழங்குநர்களின் அறிக்கைகள் உட்பட கல்வி சாரா இலக்கியங்கள்; அரசாங்க ஆவணங்கள்; கருத்துத் துண்டுகள் மற்றும் நிபுணர்களின் மதிப்புரைகள்.

**தேடல் அளவுகோல்கள் மற்றும் தகுதிக்கான அளவுகோல்கள்:** பாடத்தின் தன்மை மற்றும் முடிந்தவரை உள்ளடக்கியதாக இருக்க, மருத்துவ இமேஜிங் சாதனங்கள் அல்லது இன்பர்மேட்டிக்ஸ் துணை வகைகளுக்கு குறிப்பிட்டதாக இல்லாத பரந்த சொற்களைப் பயன்படுத்தி ஒரு தேடல் நடத்தப்பட்டது. முதன்மையாக இந்தியாவில் நடத்தப்பட்ட பணிகள், 2005 முதல் மார்ச் 2022 வரை ஆங்கிலத்தில் வெளியிடப்பட்டன, மேலும் முழு கையெழுத்துப் பிரதிகள் உள்ளவை சேர்க்கப்பட்டுள்ளன.

**ஆய்வு மதிப்பீடு மற்றும் தொகுப்பு முறைகள்:** முழு-உரை மதிப்பாய்வுக்கான சேர்க்கை அளவுகோல்களுக்கு எதிராக இரண்டு ஆசிரியர்கள் சுயாதீனமாக சுருக்கங்களைத் திரையிட்டனர் மற்றும் ஒரு மூத்த எழுத்தாளர் முரண்பாடுகளைத் தீர்த்தார். ஒரு மதிப்பாய்வாளரால் DistillerSR மென்பொருளைப் பயன்படுத்தி தரவு பிரித்தெடுக்கப்பட்டது மற்றும் இரண்டாவதாக குறுக்கு சோதனை செய்யப்பட்டது. கிரிட்டிகல் அப்ரைசல் ஸ்கில்ஸ் புரோகிராம் (CASP) சரிபார்ப்புப் பட்டியலைப் பயன்படுத்தி சார்பு மதிப்பீடு செய்யப்பட்டது.

**முடிவுகள்:** 43 அசல் கட்டுரைகள் மற்றும் 52 கல்வி சாரா பொருட்கள் இறுதியாக மதிப்பாய்வு செய்யப்பட்டன. அசல் கட்டுரைகளின் பரந்த போக்குகள்: டெலிரேடியாலஜியைப் பயன்படுத்தி இணைப்பு (n=17), மொபைல் டிஜிட்டல் இமேஜிங் யூனிட்கள் (n=9), செயற்கை நுண்ணறிவு (n=16); மொபைல் சாதனங்கள் மற்றும் ஸ்மார்ட்போன் பயன்பாடுகள் (n=7); தரவு பாதுகாப்பு (n=7) மற்றும் இணைய அடிப்படையிலான தொழில்நுட்பம் (n=2); பொது-தனியார் கூட்டாண்மை (n=9); செலவு (n=2); ஒத்திசைவு (n=19); மதிப்பீடு தேவை (n=12); மதிப்பீடு (n=4); செயல்படுத்தல் (n=2). மதிப்பாய்வில் 10 இந்திய மாநிலங்களின் தரவுகள் அடங்கும், கிராமப்புற அமைப்புகளிலிருந்து சில (n=9). டெலிரேடியாலஜி மற்றும் AI இன் பங்கு நிமோனியா, காசநோய், இன்ட்ராக்ரானியல் இரத்தப்போக்கு மற்றும் அவசர கதிரியக்கத்தில் மிகவும் நிரூபிக்கப்பட்டது.

**முக்கிய கண்டுபிடிப்புகளின் முடிவுகள் மற்றும் தாக்கங்கள்**: டெலிரேடியாலஜி, குறிப்பாக AI மற்றும் மொபைல் டிஜிட்டல் இமேஜிங் யூனிட்களுடன் இணைந்தால், கதிரியக்க நிபுணர் பற்றாக்குறையை நிவர்த்தி செய்ய முடியும் என்று கிடைக்கக்கூடிய சான்றுகள் தெரிவிக்கின்றன; கண்டறிதல் மற்றும் ஆரம்பகால நோயறிதலுக்கான மக்கள்தொகை பரிசோதனையை இலக்காகக் கொண்ட திட்டங்களை வலுப்படுத்துதல்; மற்றும் அவசர சிகிச்சை. இருப்பினும், இந்தியாவிற்குள் டெலிரேடியாலஜி நெட்வொர்க்குகளின் அளவில் போதுமான தரவு இல்லை; தர தரநிலைகள், தரவு பாதுகாப்பு மற்றும் இரகசியத்தன்மையை நிர்வகிக்கும் விதிமுறைகள்; மதிப்பீடு தேவை; செலவு; ஆரம்ப சுகாதார அமைப்புகளில் டெலிரேடியாலஜி மற்றும் புதிய தொழில்நுட்பங்களை செயல்படுத்துவதற்கான வசதிகள் மற்றும் தடைகள்; மேலும் இது தனிப்பட்ட சுகாதார விளைவுகளில் நேரடி தாக்கத்தை ஏற்படுத்தும்.

**முக்கிய வார்த்தைகள்:** டெலிரேடியாலஜி; மொபைல்-டெலரேடியாலஜி; ஆரம்ப சுகாதார பராமரிப்பு; அணுகல்; தொழில்நுட்பம்; செயற்கை நுண்ணறிவு; உலகளாவிய சுகாதார பாதுகாப்பு

**முக்கிய கண்டுபிடிப்புகள் / சிறப்பம்சங்கள்:**

1. தொலைதூர புவியியல் பகுதிகளில் இமேஜிங் கவனிப்புக்கான அணுகலை மேம்படுத்த டெலிரேடியாலஜியை செயல்படுத்துவதற்கான சாத்தியம், பலம் மற்றும் சவால்களை கிடைக்கக்கூடிய தரவு நிரூபிக்கிறது.
2. AI அல்காரிதம்கள் மீதான ஒத்திசைவு ஆய்வுகள், மார்பு ரேடியோகிராஃப்களில் பொதுவான நோய்க்குறியீடுகளை விளக்குவதற்கும் மூளை CT ஸ்கேன்களில் உள்ள மண்டையோட்டு இரத்தக் கசிவைக் கண்டறிவதற்கும் கதிரியக்கவியலாளருடன் ஒப்பிடக்கூடிய முடிவுகளைக் காட்டுகின்றன.
3. பொது-தனியார் கூட்டாண்மை (பிபிபி) மாதிரிகளில் காசநோயைக் கட்டுப்படுத்த செயற்கை நுண்ணறிவு மற்றும் மொபைல் டிஜிட்டல் எக்ஸ்ரே அலகுகளைப் பயன்படுத்துவது பற்றிய ஆரம்ப முடிவுகள் நம்பிக்கைக்குரியவை மற்றும் சுகாதார ஊழியர்களின் பற்றாக்குறையை நிவர்த்தி செய்வதற்கும் அணுகலை மேம்படுத்துவதற்கும் பெரும் ஆற்றலைக் கொண்டுள்ளன.
4. PPP மாதிரிகள் உயர்-வள குறைந்த அதிர்வெண் கதிரியக்க சேவைகளுக்காக நாடு முழுவதும் பரவலாக ஏற்றுக்கொள்ளப்பட்டுள்ளன. கிடைக்கக்கூடிய இலக்கியங்கள் இந்த மாதிரிகளின் பலம் மற்றும் பலவீனங்களை நிரூபிக்கின்றன.
5. டெலிரேடியாலஜி மற்றும் செயற்கை நுண்ணறிவு அடிப்படையிலான சேவைகள் ஆகிய இரண்டும் எக்ஸ்-ரே பிலிம்களை டிஜிட்டல் மயமாக்கும் முறைகளைப் பின்பற்றுவதன் மூலம் நாடு முழுவதும் பரவலாகப் பயன்படுத்தப்படும் அனலாக் எக்ஸ்-ரே அலகுகளைச் சேர்க்கும் வகையில் மாற்றியமைக்கப்பட்டுள்ளன.
6. ஐபாட், மொபைல் போன்கள் போன்ற மாற்று படக் காட்சி அலகுகள் மற்றும் வாட்ஸ்அப் போன்ற சமூக ஊடக தளங்கள், பல் மருத்துவ சேவைகள், குறிப்பிட்ட நோய்களின் எக்ஸ்ரே, அதிர்ச்சி மற்றும் மண்டைக்குள் இரத்தப்போக்கு போன்ற சில குறிப்பிட்ட மருத்துவ சூழ்நிலைகளில் போதுமானவை.
7. தரவு பாதுகாப்பு மற்றும் தரவு பாதுகாப்பு பற்றிய இலக்கியம் குறைவாக உள்ளது.
8.  தரவு பாதுகாப்பு, நோயாளியின் ரகசியத்தன்மை மற்றும் டெலிரேடியாலஜி நடைமுறைகளின் தரத் தரங்களை நிர்வகிக்கும் விதிமுறைகள் தெளிவாக இல்லை.
9. மதிப்பீடு, செலவு-செயல்திறன், தேவைகள் மதிப்பீடு ஆகியவற்றில் அறிவு இடைவெளிகள் உள்ளன; ஆரம்ப சுகாதாரத்திற்கான அணுகலை மேம்படுத்த டெலிரேடியாலஜி மற்றும் பிற புதிய தொழில்நுட்பங்களைப் பயன்படுத்துவதற்கான வசதிகள் மற்றும் தடைகள்.
